# Poor sleep quality and its predictors among type 2 diabetes mellitus patients attending Jimma University Medical Center, Jimma, Ethiopia

**DOI:** 10.1186/s13104-019-4531-6

**Published:** 2019-08-06

**Authors:** Tadeg Jemere, Andualem Mossie, Hiwot Berhanu, Yigizie Yeshaw

**Affiliations:** 1 Department of Biomedical Sciences, College of Health Sciences, Debre Tabor University, Debre Tabor, Ethiopia; 2 Department of Biomedical Sciences, College of Health Sciences, Jimma University, Jimma, Ethiopia; 3 Department of Medical Physiology, College of Medicine and Health Sciences, University of Gondar, Gondar, Ethiopia

**Keywords:** Sleep quality, Type II diabetes mellitus, Ethiopia

## Abstract

**Objectives:**

The aim of this study is to determine the prevalence of poor sleep quality and its associated factors among people with type 2 diabetes mellitus at Jimma University Medical Center, Jimma, Ethiopia 2018. Comparative cross-sectional study was employed on 198 participants (99 cases and 99 controls). Data were collected using Pittsburgh Sleep Quality Index (PSQI) and analyzed using SPSS version 20. Variables with *p* value ≤ 0.05 in multivariable logistic regression were treated as significant predictors of poor sleep quality.

**Results:**

The prevalence of poor sleep quality was 55.6% among people with type 2 diabetes mellitus and 32.3% among controls. Longer duration of diabetes since diagnosis (> 10 years) [AOR = 4.88 CI (1.27, 18.66) p = 0.021], co-morbid hypertension [AOR = 3.2, CI (1.16, 8.84) p = 0.025], poor glycemic control [AOR = 3.16 CI (1.2, 8.27), p = 0.02] and current khat chewing [AOR = 3.06, CI (1.04, 8.98), p = 0.042] were factors significantly associated with poor sleep quality. The prevalence of poor sleep quality was significantly higher among people with diabetes than those who didn’t have diabetes (controls). Poor sleep quality may bring about mental impairment and reduce working capacity of individuals with diabetes mellitus. Therefore, diabetes mellitus patients need to have heath education about risk factors for poor sleep quality.

## Introduction

Sleep is a basic biologic function which is essential for life [1]. It is defined as unconsciousness from which the person can be aroused by sensory or other stimuli [2]. It is the time when the body secretes many important hormones that affect growth, sleep, regulate energy, metabolic and endocrine functions [3, 4].

Good quality sleep is one of the most important things that we need to keep our body healthy which really requires adequate duration, appropriate timing and regularity, and the absence of sleep disorders [5].

People with diabetes mellitus are at greater risk of developing sleep disturbance symptoms than the general population, which may be associated with the diabetes itself or with the complications that develop as the disease progresses [6]. Diabetes Mellitus (DM) can predispose to disturbed sleep patterns and vice versa [7–10]. Pain and discomfort caused by diabetes neuropathy, Restless Legs Syndrome and nocturnal polyuria, have been proposed to contribute DM-related sleep problems [11].

Along with the growing epidemic of DM and obesity, the magnitude of sleep disturbances and deprivation has been increasing dramatically over the past decades worldwide [12]. The finding of different studies shows that the prevalence of sleep disturbances among people with type 2 diabetes ranges from 38 to 97% [9–11, 13, 14], which imposes a higher caregiver burden and impairing quality of life for both patients and their bed-partners.

Insufficient sleep is associated with many negative health outcomes and increased risk of mortality. It impairs cognitive performance, and substantially reduces workplace productivity through absenteeism [15].

According to the findings of different studies, female gender, low income, longer disease duration, poor glycemic control, and presence of hypertension increases the risk of poor sleep quality among people with type 2 diabetes [10, 16–18].

Even though DM is associated with many health problems, there is scarcity of data that determine the magnitude and determinants of poor sleep quality among people with type 2 diabetes. Therefore, we conduct this study so as to fill the above gap by determining the prevalence of sleep quality and its associated factors among people with type 2 diabetes.

## Main text

### Methods

#### Study design, area and period

Institution based comparative cross-sectional study was conducted at Jimma University Medical Center (JUMC) from April 5 to May 5, 2018. Jimma University Medical Center is one of the oldest university hospital in Ethiopia, which serves as a referral unit and training center for both medical and health science students. The Hospital serves for a total of 2594 people with type 2 diabetes during the time of data collection.

#### Source and study population

All adult Type 2 DM patients who were enrolled to JUMC and healthy individuals who came to the hospital for routine purpose were the source populations. Type 2 DM patients who were attending JUMC and age and sex matched relatively healthy individuals present at the time of data collection were the study populations.

#### Eligibility criteria

All adult cases who had complete medical records and controls who didn’t have family history of DM were included. Those people with type 2 diabetes who had diabetes ketoacidosis, psychiatric disorder, other chronic illnesses, working in night shifts in the last month, and those pregnant and lactating mothers were excluded.

#### Sample size and sampling procedure

The sample size of the study was calculated using two population proportion formula, by considering the proportion of poor sleep quality among cases (P1 = 53.4%) and controls (P2 = 29.5%) in another similar study [8], 95% confidence level and 90% power. After adding 10% non-response rate, the sample size for each group was 99, yielding the total sample size of 198. Consecutive sampling technique were used to select cases and convenient sampling technique for controls.

#### Data collection procedure

Interviewer administered questionnaire was used to collect the data. The PSQI which provides information about sleep quality over a month’s time was used for sleep quality. It is a 19-item self-report measure which has seven subscales. These subscales are added in order to determine a global sleep quality score (GSQ). The GSQ score ranges from 0 to 21, in which higher scores (PSQI > 05) indicate poor sleep quality [19]. Weight and height were measured using digital weight scale and stadiometer respectively. Blood pressure (BP) was also measured using automated digital BP monitor. Data were collected by two trained BSc nurses.

#### Operational definitions

Good glycemic control: a 3 month average fasting blood glucose measurement between 70 and 130 mg/dL.

Poor glycemic control: average blood glucose measurements on three consecutive visits is > 130 or < 70 mg/dL.

Individuals who use any of the substances such alcohol, khat, and cigarette smoking as at least once in their life time were classified as ever user. Participants who consumed psycho-stimulant substances such as alcohol, khat, and cigarette smoking at least once within the last 30 days was classified as current user.

#### Data analysis procedure

Data were checked for completeness, entered into Epi-data version 3.1 and exported to SPSS version 20.0 for analysis. Bi-variable and multivariable logistic regression analysis were done to determine the association between different explanatory variables and poor sleep quality. All variables with a p-value < 0.25 in the bi-variable analysis were entered into multivariable logistic regression model. We used p < 0.25 as a cutoff point to select candidate variables of the final model so as to improve the chances of retaining meaningful confounders. Odds ratio and its 95% confidence interval were estimated for potential predictors of poor sleep quality, which were included in the final model. A p-value ≤ 0.05 was used to declare statistically significant.

#### Data quality control

To assure data quality, pre-tested interviewer administered questionnaire was used to collect data. A 2 day training was given for data collectors regarding the purpose of the study and measurement techniques. The questioner was translated to Afaan Oromo and Amharic language and then retranslated back to English to maintain its consistency. To minimize bias, respondents were assured to keep their response confidential.

### Results

A total of 198 participants (99 cases and 99 controls) were included in the study making a response rate of 100%. The male to female ratio was 1.11. The mean age of Type 2 DM patients was 50.14 years and that of controls was 49.9 years (*p *= 0.872). More than one-third (35.9%) of respondents were in the age range of 40 to 50 years. Twenty-five percent of people with T2DM and 26.3% controls had no formal education. Majority, 83.8% of T2DM patients and 76.8% of controls were married. Regarding monthly income, the average monthly income of the controls was higher than the cases (Table 1).

**Table 1:** Socio-demographic characteristics of study participants at JUMC, Ethiopia, 2018

Study groups N = 198				
Variables	Category	Total (n = 198) N (%)	T2DM group (n = 99) N (%)	Non-D2M group (n = 99) N (%)
Age (years)	Mean ± SD		50.14 ± 11.3	49.9 ± 9.7
	29–39	38 (19.2)	20 (20.2)	18 (18.2)
	40–50	71 (35.9)	32 (32.3)	39 (39.4)
	51–60	51 (25.7)	28 (28.3)	23 (23.2)
	> 60	38 (19.2)	19 (19.2)	19 (19.2)
Sex	Male	104 (52.53)	52 (52.5)	52 (52.5)
	Female	94 (47.47)	47 (47.5)	47 (47.5)
Educational status	No formal	51 (25.8)	25 (25.3)	26 (26.3)
	Primary	59 (29.8)	27 (27.3)	32 (32.3)
	Secondary	39 (19.7)	19 (19.2)	20 (20.2)
	≥ Diploma	49 (24.7)	28 (28.3)	21 (21.2)
Marital status	Single	8 (4.04)	4 (4)	4 (4)
	Married	159 (80.3)	83 (83.8)	76 (76.8)
	Divorced	20 (10.1)	8 (8.1)	12 (12.1)
	Widowed	11 (5.55)	4 (4)	7 (7.1)
Occupation	Employed	40 (20.2)	19 (19.2)	21 (21.2)
	Merchant	21 (10.6)	8 (8.1)	13 (13.1)
	Farmer	56 (28.3)	22 (22.2)	34 (34.3)
	House wife	36 (18.2)	20 (20.2)	16 (16.2)
	Others**	45 (22.7)	30 (30.3)	15 (15.2)
	Mean ± SD		1688.58 ± 1488.96	1928.72 ± 1860.46
Monthly income	> 1000 ETB	96 (48.5)	45 (45.5)	51 (51.5)
	≤ 1000 ETB	102 (51.5)	54 (54.5)	48 (48.5)
Residence	Urban	116 (58.6)	61 (60.6)	55 (55.6)
	Rural	82 (41.4)	38 (38.4)	44 (44.4)

Others* = Guraghe, Tigre, Dawro, Welayta, Others** = Private worker, Daily laborer, unemployed, student. ETB: Ethiopian birr

#### Substance use and clinical characteristics of study participants

Thirty-eight (38.4%) of cases and 42 (42.4%) of controls chewed khat at least once in their life time. Thirty-four (34.3%) of cases and 37 (37.4%) of controls were current chewers. Regarding alcohol consumption, 30 (30.3%) of cases and 39 (39.4%) of controls had history of alcohol consumption. 9 (9.1%) of cases and 12 (12.1%) of controls smoke cigarette in their life time.

The average duration of DM since diagnosis was 6.88 years. Regarding treatment, 47.5% of T2DM patients were taking oral hypoglycemic agents. More than half (59.6%) of people with type 2 DM had poor glycemic control (FBS > 130 mg/dL) with mean FBS level of 149.97 mg/dL. Forty-six (46.5%) of type 2 diabetes had co-morbid hypertension. Seven (7.1%) of T2DM patients were diagnosed with chronic complications of DM.

The mean BMI was 24.2 for cases and 21.3 for controls. Overall, 2 (2%) of cases and 13 (13.1%) of controls had under-weight. On the other hand, 36 (36.4%) of cases and 11 (11.1%) of controls had over-weight. Eight (8.1%) of cases and 2 (2%) of controls were found to be obese. One-third (33.3%) of the cases and 3 (3%) of controls had hypertension at the time of data collection.

#### Prevalence of poor sleep quality

The overall prevalence of poor sleep quality was 55.6% for cases 95% CI (45.5, 64.5) and 32.3% for that of controls 95% CI (23.2, 41.4). The mean GSQ score was 6.58 for cases and 3.61 for controls (*p *= 0.000).

The average bed time of the participants was 10:01 pm for T2DM patients and 10:05 pm for non-diabetes individuals. The mean total time to fall asleep was 33 min for cases and 21 min for controls. The mean actual sleep time 6.2 h for T2DM group and 6.9 h for controls.

The seven components of sleep quality were also assessed. Accordingly, 16 (16.2%) of cases and 4 (4%) of controls rate their subjective sleep quality as very bad. Majority, (78.8%) of cases and 65.6% of controls had ≤ 7 h of sleep per night. The habitual sleep efficiency was < 65% in 26.3% of cases and in 6.1% of controls (Table 2).

**Table 2:** Sleep quality and its component scores among study subjects at JUMC, Ethiopia, 2018

Study participants (N = 198)			
Variables	Category	T2DM group N (%)	Non-diabetic group N (%)
Sleep duration	> 7 h	21 (21.2)	35 (35.4)
	6–7 h	53 (53.5)	45 (45.5)
	5–6 h	14 (14.1)	17 (17.2)
	< 5 h	11 (11.1)	2 (2)
Sleep latency	Never (0)	25 (25.3)	38 (38.4)
	< 1 times a week (1)	50 (50.5)	44 (44.4)
	1–2 times a week (2)	20 (20.2)	14 (14.1)
	≥ 3 times a week (3)	4 (4)	3 (3)
Sleep efficiency	≥ 85%	22 (22.2)	57 (57.6)
	75–84%	40 (40.4)	28 (28.3)
	65–74%	11 (11.1)	8 (8.1)
	< 65%	26 (26.3)	6 (6.1)
Day time dysfunction	Never (0)	38 (38.4)	72 (72.7)
	< 1 times a week (1)	52 (52.5)	26 (26.3)
	1–2 times a week (2)	8 (8.1)	1 (1)
	≥ 3 times a week (3)	1 (1)	0
Sleep disturbance	Never (0)	3 (3)	23 (23.2)
	< 1 times a week (1)	89 (89.9)	76 (76.8)
	1–2 times a week (2)	6 (6.1)	0
	≥ 3 times a week (3)	1 (1)	0
Use of sleep medication	Never (0)	86 (86.9)	95 (96)
	< 1 times a week (1)	12 (12.1)	4 (4)
	1–2 times a week (2)	1 (1)	0
Subjective sleep quality	Very good (0)	25 (25.25)	59 (59.6)
	Fairly good (1)	37 (37.4)	36 (36.4)
	Fairly bad (2)	21 (21.2)	4 (4)
	Very bad (3)	16 (16.15)	0

#### Predictors of poor sleep quality

The independent predictors of poor sleep quality among T2DM patients were: duration of DM > 10 years [AOR = 4.88 CI (1.27, 18.66) *p *= 0.021], presence of co-morbid hypertension [AOR = 3.19, CI (1.16, 8.84) *p *= 0.025], poor glycemic control [AOR = 3.16 CI (1.2, 8.27), *p *= 0.02] and current khat chewing [AOR = 3.058, CI (1.04, 8.98), *p *= 0.042] (Table 3).

**Table 3:** Bivariable and multivariable logistic regression model of factors independently associated with poor sleep quality among T2DM clients at JUMC, Jimma, Ethiopia, 2018, (N = 99)

Variables	Poor sleep quality		OR (95% CI)	
	Yes N (%)	No N (%)	COR	AOR
Duration of DM in years				
1–4	15 (39.5)	23 (60.5)	1	1
5–10	25 (61)	16 (39)	2.4 (0.9, 5.9)	1.7 (0.6, 4.7)
> 10	15 (75)	5 (25)	4.6 (1.4, 15.3)	4.88 (1.3, 18.7)*
Co-morbid HTN				
Yes	32 (69.6)	14 (30.4)	2.9 (1.3, 6.8)	3.2 (1.2, 8.8)*
No	23 (43.4)	30 (56.6)	1	1
Glycemic control				
Good	14 (35)	26 (65)	1	1
Poor	41 (69.5)	18 (30.5)	4.2 (1.8, 9.9)	3.2 (1.2, 8.3)*
Current khat chewing				
Yes	22 (64.7)	12 (35.3)	1.8 (0.7, 4.2)	3.0 (1.02, 8.9)*
No	33 (50.8)	32 (49.2)	1	1

*p-*value* < 0.05, COR: Crude Odds Ratio

### Discussion

This study revealed a high prevalence of poor sleep quality among T2DM patients (55.6%) than non-diabetes (controls) (32.3%). This is in agreement with studies conducted in USA (55%) [20], Korea (49%) [21], Kenya (53.4%) [8], and Trinidad and Tobago, (63.9%) [22]. However, this finding is higher than the finding of another similar study in Iran (38%) [9]. The result of this study is lower than studies conducted in Turkey (77.4%) [16], Saudi Arabia (72%) [17], USA (84%) [14] and Sudan (97.1%) [13]. The possible reason for this discrepancy might be difference in PSQI cutoff point, socioeconomic demands and recruitment criteria.

This study also revealed that, prevalence of poor sleep quality differed significantly according to disease duration and glycemic control. Type 2 DM patients who had longer disease duration (> 10 years) and/or poor glycemic control (FBS > 130 mg/dL) had higher risk of poor sleep quality than those with shorter duration of disease and/or good glycemic control. This finding is in line with the study conducted in China [10] and Saudi Arabia [17]. This might be related to the occurrence of diabetes complications as duration of diseases gets longer. Moreover, diabetes patients with poor glycemic control will develop nocturia, which leads to frequent awakenings, to result in poor sleep quality [23].

Those T2DM patients with co-morbid hypertension had increased risk of poor sleep quality than who didn’t have hypertension. This is in line with the study conducted in Brazil [23] and Turkey [16]. This might be due to the increased risk of emotional disorders such as anxiety and depression among DM cases with comorbid hypertension compared to DM cases only, which are the risk factors of poor sleep quality [24].

Current khat chewers’ participants had increased risk of poor sleep quality than non-chewers. This finding agrees with the study conducted in Jimma town [25]. This could be the result of sympathomimetic effects of khat [26]. Khat (Catha edulis) is a stimulant with effects similar to amphetamine, because the main active ingredient of khat (cathinone), is an amphetamine like substance [26]. Effects of cathinone are mediated through decreased dopamine uptake by nerve terminals, increased dopamine release and inhibition of monoaminoxidase [25, 26]. Those all processes might lead to poor sleep quality, due to persistent stimulation of post synaptic neurons following high level of dopamine in synaptic cleft.

Increased secretion of catecholamine’s might also further increase blood glucose level by activation of glycogenolysis in skeletal muscles and liver, which might further exacerbate the glycemic control to increase poor sleep quality. Moreover, pesticides like DDT sprayed on khat, might inhibit pancreatic secretary activity by reducing calcium permeability of β cells, which would also elevate blood glucose level and result in an increased risk of poor sleep quality [25–29].

## Limitations of the study

First, the sample size was limited. Second, glycemic control was measured with FBS level rather than the glycated hemoglobin due to budget constraint. Despite these limitations, our study clearly showed the magnitude of poor sleep quality and its predictors in our country, which is not well investigated so far.

## Data Availability

The authors confirm that all data underlying the findings are fully available without restriction. All relevant data are within the manuscript.

## Abbreviations

AOR: Adjusted Odds Ratio
BMI: Body Mass Index
BP: blood pressure
CI: confidence interval
GSQ: global sleep quality score
T2DM: type 2 diabetes mellitus
